# Direct Evidence for Vision-based Control of Flight Speed in Budgerigars

**DOI:** 10.1038/srep10992

**Published:** 2015-06-05

**Authors:** Ingo Schiffner, Mandyam V. Srinivasan

**Affiliations:** 1 Queensland Brain Institute; 2 School of Information Technology and Electrical Engineering & ARC Centre of Excellence in Vision Science; 3 University of Queensland, St Lucia, QLD 4072, Australia

## Abstract

We have investigated whether, and, if so, how birds use vision to regulate the speed of their flight. Budgerigars, Melopsittacus undulatus, were filmed in 3-D using high-speed video cameras as they flew along a 25 m tunnel in which stationary or moving vertically oriented black and white stripes were projected on the side walls. We found that the birds increased their flight speed when the stripes were moved in the birds’ flight direction, but decreased it only marginally when the stripes were moved in the opposite direction. The results provide the first direct evidence that Budgerigars use cues based on optic flow, to regulate their flight speed. However, unlike the situation in flying insects, it appears that the control of flight speed in Budgerigars is direction-specific. It does not rely solely on cues derived from optic flow, but may also be determined by energy constraints.

While considerable effort has been devoted to investigating long-range navigation in a number of bird species^1^, we know relatively little about whether, and if so how, birds use their vision to guide moment-to-moment flight. The past few decades have witnessed substantial progress in our understanding of how flying insects use visual information to stabilise their flight, control their flight speed, avoid collisions with obstacles, estimate distance flown, and perform smooth landings^2^,^3^. Meanwhile, evidence has been slowly accumulating to suggest that birds may also use similar principles to guide their flight, at least with respect to some visually mediated behaviours^4^,^5^,^6^,^7^. Some examples of convergent principles are the detection of edges for landing Budgerigars:^7^, Bees:^8^ and the use of optic flow cues for flying safely through narrow passages Budgerigars:^4^, Bees:^9^,^10^. However, compared to insects, our present understanding of visually guided flight in birds is relatively sparse.

Here we investigate the control of flight speed in Budgerigars, and the role that their vision may play in this context. Budgerigars, native parakeets of Australia inhabiting dry scrublands, have a seemingly effortless capability to navigate through cluttered environments and to avoid obstacles and predators, by tailoring their flight appropriately^11^. Budgerigars are ideally suited for this study because they are relatively easy to maintain and are willing to perform flights without much need for training or incentives such as food rewards.

We video-recorded the flight trajectories of 11 birds, flying along a 25 m long purpose-built tunnel (See Fig. 1). Stationary or moving patterns (vertical black and white stripes) were projected onto the side walls, allowing direct manipulation of the optic flow experienced by the birds in flight (see ‘Experimental Procedures’). Because the stripes were oriented perpendicular to the bird’s flight direction, they provided strong optic flow cues. We tested 6 different conditions, i.e. 3 different speeds of pattern motion, with the motion being directed either along the bird’s flight trajectory or against it. In each case we also recorded flights under a control condition, in which the patterns were stationary (see ‘Experimental Procedures’). A total of 528 flights were recorded. Each flight was reconstructed in 3-D and analysed to measure the mean flight speed, as described in ‘Experimental Procedures’. We examined whether, and if so, in what way the birds’ flight speeds were affected by the optic flow that was induced in their eyes by the stationary or the moving patterns.

## Results

We began by measuring the average flight speed of the birds when they flew through the tunnel when stationary stripes were displayed on both walls (control condition). The 3D speed under control conditions, averaged over all pairs of frames, all flights and birds, was 6.31 ± 0.17 m/s. This figure was only slightly higher than the average axial speed of 6.14 ± 0.19 m/s, indicating that the birds were flying predominantly along the length of the tunnel. As the birds’ motion was directed primarily along the longitudinal axis of the tunnel, it is safe to assume that the birds where performing the intended task, i.e. fly to the other end of the tunnel, and that they were not under any kind of duress, which would be indicated by motion directed primarily upwards in search of an escape hole or a high perch. During control trials as well as experimental trials, the birds flew at an average height of 1.38 m, which is substantially lower than the height of the tunnel’s ceiling (2.4 m).

To eliminate potential effects from variable track lengths, we used for our comparisons the 3D speed measured over a fixed, pre-defined segment of the tunnel that offered the best tracking coverage (see ’Experimental Procedures’). The average flight speed measured over this segment was 6.28 ± 0.11 m/s. In addition we also estimated both intra-individual variation over all trials of each individual, which was on average ± 0.56 m/s (SD), and inter-individual variation over all birds, which was ± 0.85 m/s (SD), suggesting that not only were individual birds maintaining fairly constant speeds throughout all trials, but also that all birds were flying at relatively similar speeds. As shown in Fig. 2, the speed measured in the control trials was more or less constant – the standard deviation averaged over all individuals was 0.21 m/s - throughout the selected tunnel segment over which it was measured, suggesting that, at least within this segment of the tunnel the speed of the birds’ flight was not affected by acceleration during take-off, or deceleration during landing.

Next, we examined the effect of pattern motion on flight speed. The results are summarized in Fig. 3, which shows the mean change in flight speed relative to the control (stationary patterns), for each of the six conditions in which the patterns were in motion. The birds increase their flight speed when the patterns move in the direction of their flight. The higher the pattern speed, the larger the flight speed. However, the increase in flight speed is smaller than the increase in pattern speed. The blue bars in Fig. 3 indicate the flight speeds that the birds should exhibit if they were to change their flight speeds by amounts that matched the changes in the pattern speed, i.e. if they were holding the image velocity constant. This implies that the angular velocity of the image of the patterns, as experienced by the bird’s visual system, decreases from a mean value of 514 ± 66 deg/s for flights with the stationary patterns, to a mean value of 463 ± 74 deg/s for flights with the patterns moving at the maximum forward speed of 1.0 m/s. (Fig. 4). On the other hand, pattern motion in the backward direction evokes almost no change in flight speed compared to the stationary control. At the highest pattern speed (-1.0 m/s), the birds slow down by a small, but statistically non-significant amount (ca. 0.10 m/s; Fig. 3). Consequently, for backward pattern motion, the birds experience image angular velocities that increase sharply as the speed of the pattern is increased (Fig. 4).

In walking humans, it has been found that changes in the experienced optic flow result in an adjustment of stride length and cadence e.g.^12^,^13^,^14^. Here we investigated whether the wing beat of birds was affected by changes in optic flow. For this purpose, we compared the flights under conditions of high pattern velocity, i.e. −1 m/s and 1 m/s, with their respective controls. In particular we examined whether the average wing-beat duration during normal flapping flight, and the amount of time spent in undulating flight (flap-bounding or flap-gliding),^15^ were affected by the changes in pattern velocity.

The results are summarised in Table 1. When the birds experienced decreased optic flow (i.e. when the pattern was moving in the flight direction- Experiment A), there was a significant decrease in the wingbeat duration, i.e. an increase in wingbeat frequency, as well as a decrease in the total number of wing-beats. This results in a higher average distance traversed per wingbeat, but not necessarily a higher flight speed. The duration of the undulating phases was unaffected. However, the birds showed a tendency to spend more time undulating relative to the total duration of the flight.

On the other hand, when the birds experienced increased optic flow, i.e. when the pattern was moving against the flight direction - Experiment B), there was no significant change in wing-beat duration, and a small, but statistically significant increase in the number of wing-beats. The amount of time spent undulating was unaffected, as was the amount of time spent undulating relative to the duration of the flight.

In summary, we find that the changes (or lack thereof) in the wing kinematics that are elicited by forward and backward pattern motion mirror the changes (or lack thereof) in flight speed – forward pattern motion elicits changes in the flight speed as well as the wing kinematics, whereas backward pattern motion does not.

## Discussion

Our findings reveal that changes of flight speed are elicited only by forward motion of the patterns. This is consistent with the observation that, to a first approximation, the birds display changes in the gross kinematics of their wing movements only when the patterns move in the direction of flight, and not when they move against it. Our results indicate that forward pattern motion elicits at least three changes in the wing-beat pattern (i) lower wing-beat duration; (ii) lower total number of wing-beats; and (iii) larger proportion of time spent undulating. It is likely that a quicker wing-beat produces a greater acceleration of the air behind the wing and generates a larger thrust, propelling the bird over a larger distance per wing-beat, thus increasing its flight speed and requiring a smaller number of wing-beats to traverse the tunnel. In addition, the relative increase in time spent undulating, means a reduction in the total number of wing-beats required to traverse a given distance, and may further boost flight speed if undulating generates higher flight speeds than does normal flapping. Indeed, many studies have suggested that flap-bounding can achieve higher flight speeds at lower energy cost^15^,^16^,^17^,^18^,^19^. It is, of course, possible that forward pattern movement elicits additional changes in wing motions that we have not measured, such as changes in the articulation of the wings, or in the amplitude of the wing stroke.

To summarise, the finding that the birds alter their wing kinematics (as described above) only when the pattern moves in the same direction as the bird’s flight, but not when it moves in the opposite direction, is congruent with the observation that the birds change (increase) their flight speed only in the former case, and not in the latter.

When honeybees are flown in tunnels with moving patterns on the walls, they increase or decrease their flight speed according to whether the pattern is moved in or against the flight direction, and by an amount that was nearly equal to the speed of the pattern. As a consequence, the bees experience image angular velocities that are more or less constant at a value of 250-320 deg/s, irrespective of the speed or the direction of pattern motion^20^,^21^. Indeed, it has been postulated that bees regulate the speed of their flight by holding constant the image velocity that is experienced by their visual system^20^,^21^.

Budgerigars seem to behave somewhat differently. With forward pattern motion the birds fly faster, but not by an amount that is sufficient to maintain the image velocity at the stationary-pattern value. Thus, the image velocity decreases slightly, but steadily as the patterns move faster. It would appear that, in this condition, the birds are compensating partially, but not completely, for the forward motion of the pattern. With backward pattern motion, on the other hand, the birds show almost no attempt to compensate for the pattern motion: the flight speed remains more or less constant at the control value. During flights with stationary patterns the average image velocity experienced by the birds is 514 ± 66 deg/s in the present study (as calculated from the mean flight speed of 6.28 ± 0.11 m/s) and ca. 400 deg/s in the study of Bhagavatula *et al.*^4^. Interestingly, these figures are not very different from the values that honeybees attempt to hold in their flights (250-320 deg/s, as mentioned above).

Why are Budgerigars different, in some respects, from bees ? While it is premature to provide a definitive answer, one reason may be that, in the case of birds, flight speed is governed by additional constraints such as energy consumption. For Budgerigars it has been found that the oxygen consumption and respiratory rate are U-shaped functions of speed, with a minimum occurring at about 10 m/sec^22^,^23^. Such U-shaped functions have also been measured or theoretically predicted for other birds, for example pigeons, mallards, pheasants, doves, cockatiels and magpies^24^,^25^. For most birds, unlike insects^26^,^27^,^28^, this U-shaped curve rises more sharply at speeds lower than the optimum, compared to speeds higher than the optimum; and hovering is energetically very expensive^22^,^23^,^24^,^25^. This asymmetry becomes even more pronounced if the power curves are re-plotted to examine how the ratio of power to flight speed (which is a measure of the energy consumption per unit distance flown) varies with flight speed. The asymmetry may explain, at least in part, why the birds are amenable to increasing their flight speed above the control-condition value (which is closer to their energy-optimum speed), but not to decreasing it below this value.

Overall, the speed of the Budgerigars in the flight tunnel is considerably lower than their normal cruising speed, which has been estimated to be approximately 9.75 m/s^22^. This reduced speed is a common observation in flight tunnel experiments^4^,^11^. The reason for this is not entirely clear, but it is possible that the speed at which the birds fly is influenced not only by optic flow, but by other factors as well. One factor could be the relatively short length of the flight, i.e. the birds may not even attempt to reach cruising speed because of the restricted length of the tunnel. Another possibility is that the birds also respond to the extent of visual clutter in the environment. When flying in an open field, for example, salient visual features and the optic flow that they generate would be relatively sparse, compared to the situation in the flight tunnel, which would be akin to flying through an alleyway of trees that generates a dense optic flow field, causing the birds to fly more cautiously and slowly. If the high density of the visual texture indeed affects the birds’ behaviour in this way, it could also explain their reduced reactions to the changes in optic flow.

Another possibility is that, the birds, although flying much slower than the ‘ideal’ energy-efficient speed, are actually flying at a speed that is energy efficient for the conditions of the experiment, which involve flights that are likely to be much shorter than typical flights in a natural outdoor environment. It is important to note that wind tunnel studies on Budgerigars have almost always been restricted to continuously flapping flight, as these birds rarely show flap-gliding or flap-bounding behaviour when wearing oxygen masks^15^. Both types of undulating flight behaviour can, in theory, reduce energy cost during flight^15^,^16^,^17^,^18^,^19^. Therefore it is conceivable that the undulating flight behaviour observed in our experiments enables energy-efficient locomotion at a speed that is lower than the ‘classical’ optimum speed of 9.75 m/s.

The use of optic flow to regulate flight speed has at least two potential payoffs. First, head winds and tail winds are automatically compensated for if the flight thrust is adjusted to hold the magnitude of the optic flow constant^29^; second, flight speed is automatically reduced to a safer value when flying through narrow passages or cluttered environments^20^,^30^. It appears that Budgerigars exhibit a partial tendency to use optic flow in this way, in that they respond to changes of optic flow, by altering their flight speed to counter such changes - provided that the new speed does not take the bird completely outside its flight envelope, and is compatible with other constraints such as the total length or duration of the flight.

### Experimental Procedures

#### Ethics Statement

All experiments were carried out in accordance with the Australian Law on the protection and welfare of laboratory animals and the approval of the Animal Experimentation Ethics Committees of the University of Queensland, Brisbane, Australia.

#### Experimental Setup

The experiments were conducted in a purpose-built bird flight tunnel (height: 2.40 m, width: 1.40 m length 21.6 m). 11 male Budgerigars were trained to fly down the tunnel. Stationary or moving black and white vertical stripes, with a linear stripe period of 23.5 cm and an angular period of 18.55 deg (estimated assuming flight along the tunnel axis), were projected on the side walls of the tunnel. The striped patterns were created using ten computer-controlled data projectors, 5 mounted on either side of the tunnel such that the moving image was the same on both walls. Adjusting the speed and direction of the pattern enabled direct manipulation of the induced optic flow experienced by the birds. Two synchronised high-speed video cameras, running at a frame rate of 125 fps, were used to capture the birds’ flights in stereo, for subsequent reconstruction of the flight trajectories in 3-D. Birds flew in 6 treatment conditions (3 different pattern speeds − 1 m/s, 0.5 m/s or 0.25 m/s - moving either in or against the direction of flight) and one control condition (stationary patterns). Experiments for each treatment condition were conducted on separate days to minimise any effects of fatigue or stress on the birds’ flight performance – a bird was never flown more than 10 times on any given day). On each day birds had to perform 4 flights in one of the 6 experimental conditions and 4 flights in the control condition, to account for daily variation in the birds performance. Before each session and before the start of the actual experiment, the birds were made to perform one additional training flight in order to acquaint them with the tunnel and the experimental paradigm. This initial flight was not included in the analysis of the data. A total of 528 flights were filmed and analysed. (11 birds, with 6 stimulus conditions and 4 flights per stimulus condition, representing a total of 264 flights, plus the same number of control flights). In the experimental trials, the pattern movement was always initiated after the bird had taken off from its perch.

#### Video Recording and Tracking

**Figure 1:**
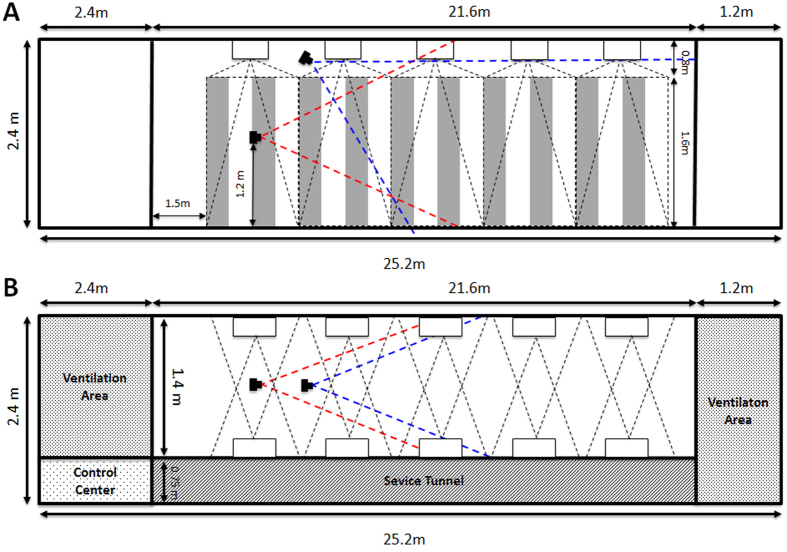
A and B: Side elevation (A) and plan (B) view of the flight tunnel. The red and blue dashed lines indicate the fields of view of the cameras. The positions of the projectors are indicated by the open rectangles and their projections by the black dashed lines. Note, figure is not to scale.

**Figure 2:**
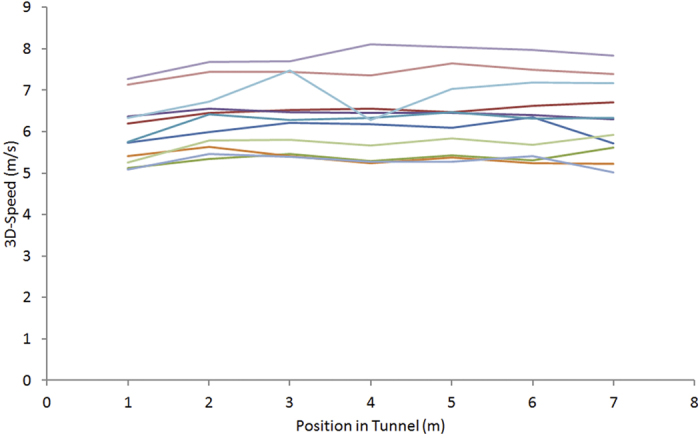
Average 3D Flight speed. Flight speed along the tunnel’s longitudinal axis, shown for each of the 11 birds averaged over all control trials, during which the gratings were stationary.

**Figure 3:**
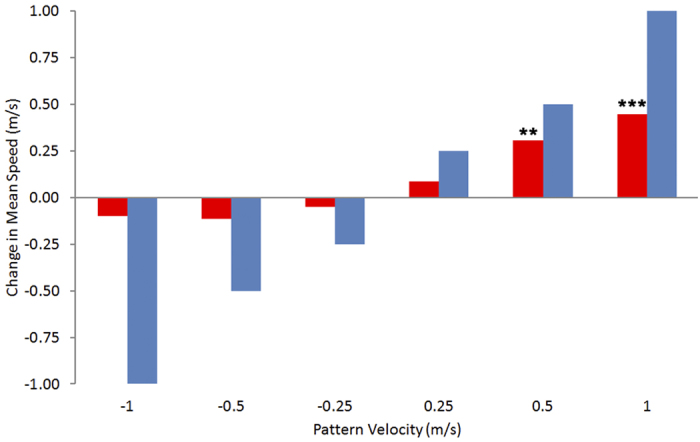
Mean change in flight speed. Change in flight speed induced by the moving patterns (red bars), relative to the flight speed prevailing for the stationary patterns (control), for each of the six pattern velocities (abscissa). Significant deviations from the control, as identified by a paired t-test, are indicated by asterisks below the bar, with ***: p < 0.001 and **: p < 0.01). The blue bars indicate the expected flight speeds if the birds were to perfectly compensate for the grating motion.

**Figure 4:**
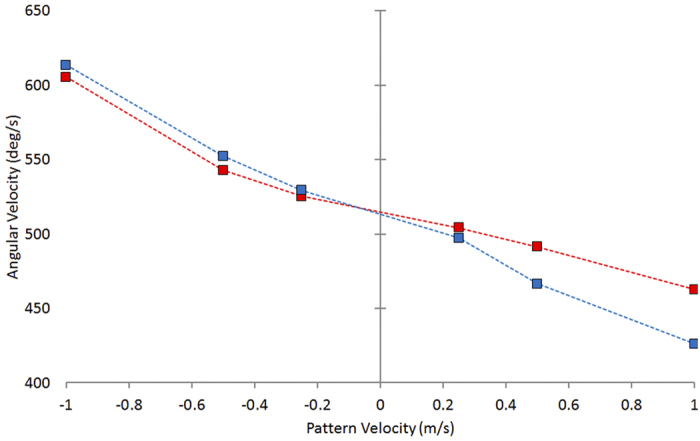
Mean image angular velocities. Image angular velocities, as experienced by the birds’ visual systems during flight in the presence of patterns moving at each of the 6 indicated velocities (red squares), compared with the theoretically expected change in image angular velocity if the birds had ignored the pattern motion and maintained the same flight speed as in the respective control experiments (blue squares). The image angular velocities were calculated as described in ‘Experimental Procedures’.

Two synchronised high-speed video cameras (Motion Pro) were used, one positioned with its optical axis aligned with the long axis of the tunnel and the other mounted on the ceiling with its optical axis tilted downwards at an angle as illustrated in Fig. 1. The cameras shared a field of view as indicated by the area defined by the red and blue dashed lines, within which the bird’s flight trajectories could be tracked in stereo. The section of the tunnel in which the birds could be tracked most reliably was determined to be the region from 8.8 m to 15.8 m along the tunnel length.

The stereo camera system was calibrated using Jean-Yves Bouguet’s Camera Calibration Toolbox for Matlab (http://www.vision.caltech.edu/bouguetj/calib_doc/index.html). Approximate average errors for the 3D reconstruction, as estimated by the toolbox, are ± 4 mm (x-axis), ± 8 mm (y-axis) and ± 19 mm (z-axis). Videos recorded by the two cameras were tracked using an automatic tracking algorithm developed in-house, allowing reconstruction of the trajectory of the bird’s centre of gravity in 3D.

#### Analysis and Statistical Evaluation

After 3D reconstruction we calculated the 3D speed for each one meter segment along the tunnel to investigate the effects of the moving patterns, as well as the mean 3D speed calculated over all data-points and the mean axial speed over all data points. For the detailed analyses we calculated the average 3D speed for each bird and each experimental condition over a fixed longitudinal tunnel segment of 7 m length (from 8.8 m to 15.8 m) This segment was chosen as it was the longest continuous segment over which the automatic tracking was accurate and reliable. These averages where then used for a comparison – using paired T-tests- between the experimental trials and the respective control trials conducted on the same day. This was done to eliminate influence of the birds’ daily performance on the comparison. In addition to this, we performed a detailed analysis of the birds’ wing-beat characteristics for the + 1 m/s and −1 m/s pattern velocities and the respective controls, to investigate if and how increased pattern velocity affected the wing-beat of the birds. Through visual inspection of the videos we determined (a) the wing-beat duration, defined as the time in ms from the start of one down-stroke to the start of the next down-stroke, and (b) the duration of flap-bounding or flap- gliding phases, for simplicity defined as all wing-beats lasting 120 ms or longer.

The angular velocities experienced by the birds during the flights in the various conditions were estimated as (180/pi)2*(V_b_ – V_p_)/W, where V_b_ is the axial bird speed (m/sec), V_p_ is the pattern velocity (m/sec, positive values representing pattern motion in the direction of bird flight), and W is the width of the tunnel. The birds were assumed to fly approximately equidistantly from the two walls (i.e. at a distance of W/2 from each wall), as is typically the case^4^.

## Additional Information

**How to cite this article**: Schiffner, I. and Srinivasan, M. V. Direct Evidence for Vision-based Control of Flight Speed in Budgerigars. *Sci. Rep.* **5**, 10992; doi: 10.1038/srep10992 (2015).

## Figures and Tables

**Table 1:** Wing-beat Characteristics. Comparison of various flight parameters when the pattern was moving at the highest speed in (+1) or against (−1) the flight direction, with a corresponding set of control measurements when the pattern was stationary.

	**Moving pattern (+1)**		**Stationary control**		**Statistical comparison**	**Moving pattern (-1)**		**Stationary control**		**Statistical comparison**
	**Avg.**	**Std. Dev.**	**Avg.**	**Std. Dev.**	**p (T-Test)**	**Avg.**	**Std.Dev.**	**Avg.**	**Std. Dev.**	**p (T-Test)**
WB-**Dur. (ms)**	60	3	62	4	0.030	60	4	60	3	0.608
WB-No.	19	5	21	6	0.006	20	5	19	5	0.038
FB-Dur.(ms)	699	125	687	103	0.700	751	156	738	141	0.744
FB-Perc.	44%	12%	40%	11%	0.067	44%	13%	44%	11%	0.893

Shown are the average duration of the wing beat (WB-Dur.), the total number of wing beats (WB-No.), the average amount of time spent flap-bounding per flight (FB-Dur.), and the relative amount of time spent flap-bounding (FB-Perc.). The p values represent the results of two-tailed paired T-tests to check for significant differences between the parameter values measured in the tests with the moving patterns, and the corresponding stationary controls. Note that the values for the stationary controls are not the same in the two sets, because separate control trials were conducted prior to each experiment with a moving pattern. This was done to account for daily variation in the birds’ performance, as experimental conditions were conducted on different days (see ‘Experimental Procedures’).
